# Effects of a small molecule R-spondin-1 substitute RS-246204 on a mouse intestinal organoid culture

**DOI:** 10.18632/oncotarget.23721

**Published:** 2017-12-26

**Authors:** Myeong-Ok Nam, Soojung Hahn, Joo Hyun Jee, Tae-Sun Hwang, Ho Yoon, Dong Hyeon Lee, Min-Soo Kwon, Jongman Yoo

**Affiliations:** ^1^ Department of Microbiology and School of Medicine, CHA University, Seongnam-si, Gyeonggi-do 13488, South Korea; ^2^ Institute of Basic Medical Sciences, School of Medicine, CHA University, Seongnam-si, Gyeonggi-do 13488, South Korea; ^3^ Department of Anatomy, School of Medicine, CHA University, Seongnam-si, Gyeonggi-do 13488, South Korea; ^4^ Department of Physiology, School of Medicine, CHA University, Seongnam-si, Gyeonggi-do 13488, South Korea; ^5^ Department of Pharmacology, School of Medicine, CHA University, Seongnam-si, Gyeonggi-do 13488, South Korea

**Keywords:** intestinal organoid, enteroid, R-spondin-1, RS-246204: Lgr5

## Abstract

Organoids, a multi-cellular and organ-like structure cultured *in vitro*, can be used in a variety of fields such as disease modeling, drug discovery, or cell therapy development. When organoids derived from Lgr5 stem cells are cultured *ex vivo*, recombinant R-spondin-1 protein should be added at a high concentration for the initiation and maintenance of the organoids. Because the addition of large amounts of R-spondin-1 greatly increases the cost of organoids, the organoids grown with R-spondin-1 are not practical for large-scale drug screening and for the development of therapeutic agents. In this study, we tried to find a R-spondin-1 substitute compound that is able initiate small intestinal organoids without the use of the R-spondin-1 protein; thus, using organoid media that each included one compound from among an 8,364 compound library instead of R-spondin-1, we observed whether organoids were established from the crypts of the small intestine. As a result, we found one compound that could promote the initial formation and growth of enteroids in the medium without R-spondin-1 and named it RS-246204. The enteroids grown with RS-246204 had a similar differentiation capacity as well as self-renewal capacity as the enteroids grown with R-spondin-1. Furthermore, the RS-246204-derived enteroids could successfully produce the forskolin induced swelling and the organoid based epithelial to mesenchymal transition model. This compound could be used for developing a cost-efficient culturing method for intestinal organoids as well as for exploring Lgr5 signaling, intestinal stem cell physiology and therapeutics for GI tract diseases.

## INTRODUCTION

An organoid is a group of cells cultured *in vitro*, which has a cell population, structure and organ specific function similar to organs [1]. Recent advances in understanding stem cell physiology and culture technology have enabled the construction of organoids derived from various organs and tissues [2]. Organoids can be used in a variety of fields such as pathophysiology through disease modeling or drug discovery or as a cell source for cell therapy development [3]. Organoids derived from specific organs can reproduce the normal physiology of the organ in a similar manner to the *in vivo* environment. For example, it is possible to model a variety of diseases producing intestinal fibrosis models by treating the organoid with several cytokines [4] as well as colorectal cancer models by introducing cancer related mutations via CRISPR/Cas9 [5] and inflammatory bowel disease (IBD) model using samples derived from patients [6, 7]. These models can provide a good platform for studying pathophysiology, screening drug efficacy, and testing drug toxicity. In addition, the organoid can restore the damaged intestinal epithelium when injected into an animal model of IBD [8–10]. The organoid may also be used to develop therapeutic agents to regenerate damaged tissue.

The methods for establishing organoids can be classified as starting from pluripotent stem cells or tissue resident stem cells. Current organoid culture techniques are capable of producing organoids from stem cells isolated from the epithelium of the digestive organs, including the gastrointestinal tract such as the stomach [11], small intestine [12], colon [13, 14], and accessory organs such as the liver [15] and pancreas [16]. The tissue-resident stem cells that form the organoids originating from the digestive organs commonly express leucine-rich repeat-containing G-protein-coupled receptor5 (Lgr5) [17] and the modulation of Lgr5 mediated Wnt signaling by R-spondin-1, which is a ligand for Lgr5, is essential for the self-renewal of stem cells [18, 19]. When organoids from Lgr5 stem cells are established *ex vivo*, recombinant R-spondin-1 protein should be added at a high concentration of 1 μg/mL to initiate organoids [20]. Because the addition of large amounts of recombinant R-spondin-1 protein reduces the cost efficiency, the organoids produced by supplementation of R-spondin-1 are impractical in studies requiring large amounts of organoids such as high throughput screening and development of therapeutic agents. As an alternative, a culture method using an R-spondin-1 enriched medium conditioned from R-spondin-1 expressing a stable cell line can save costs in organoid production, but it involves a variety of ingredients other than R-spondin-1, resulting in characteristic changes in the organoid. Such undefined components can cause interaction with drugs and biomolecules making it difficult to use them as screening or therapeutic agents. A low cost-effectiveness is a major obstacle in the development of therapeutic agents for regenerative medicines by high throughput screening based on organoids.

In this study, to develop a method for culturing organoids without recombinant R-spondin-1 protein, we sought to identify compounds capable of establishing organoids without R-spondin-1 supplementation. This not only enables the cost-efficient expansion of organoids, which can contribute to the mass preparation of organoids that can be used for clinical applications and drug screening, but also helps to study the physiology and signaling of Lgr5 stem cells.

## RESULTS

### Identification of a small molecule R-spondin-1 substitute in the mouse intestinal organoid culture

**Figure 1:**
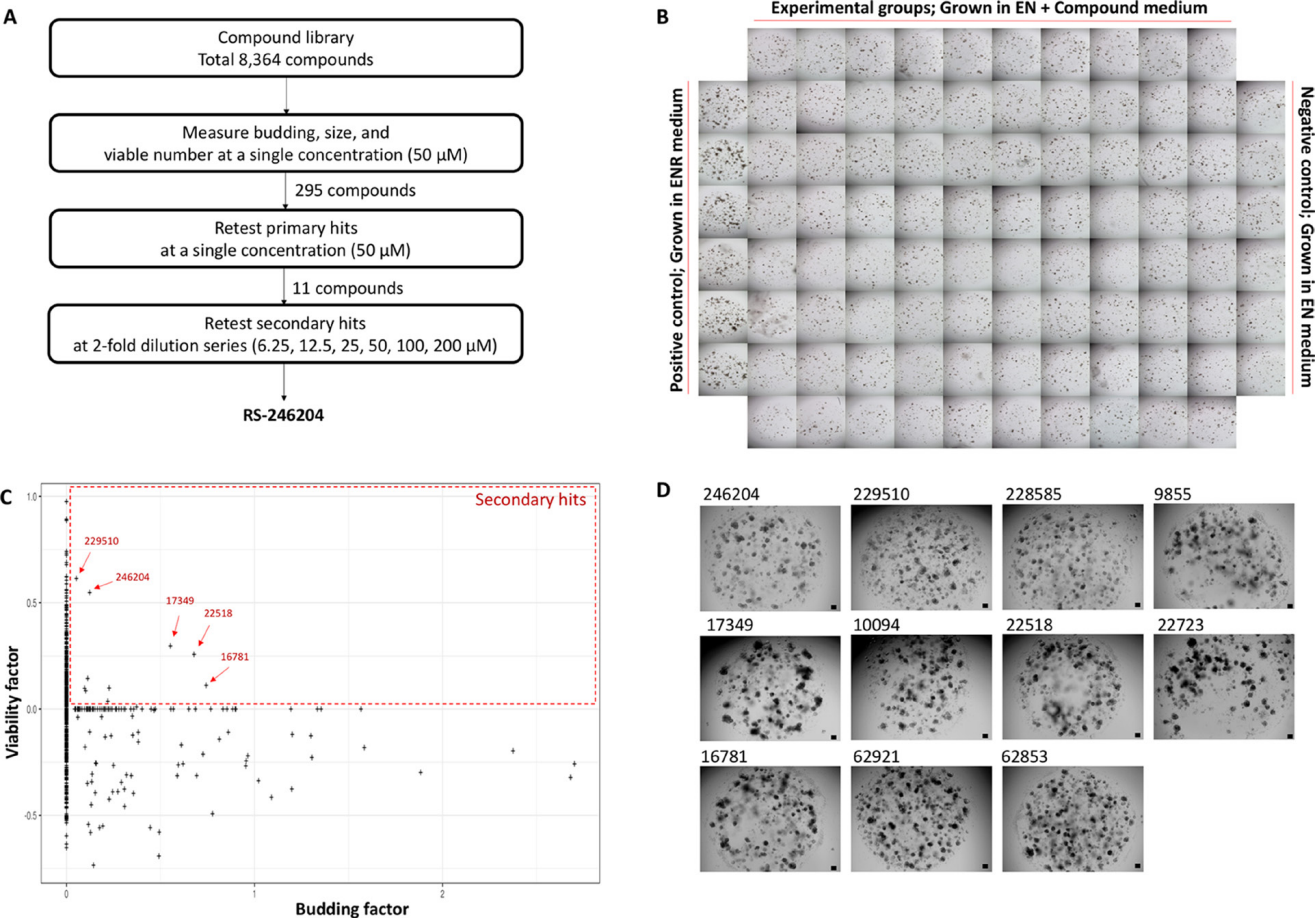
Screening to find a small molecule R-spondin-1 substitute (**A**) Schematic flowchart of the screening strategy. (**B**) Specific examples of plate configurations used in the screening. (**C**) Second retest results based on viable and budding factors for primary hits. The eleven compounds belonging to the red are the secondary hits. (**D**) A representative photograph of the result of incubating for 4 days in EN media containing the 11 compounds selected as secondary hits. Bars, 100 μm.

A chemical library with 8,364 small molecules was used to find a compound that could establish mouse small intestinal crypts into enteroids without R-spondin-1 supplementation in organoid growth medium (Figure 1A). Thus, 5 μM of each compound was added to the enteroid culture medium without R-spondin-1 supplementation (EN medium) and incubated 4 days. Crypts grown in the enteroid culture medium with R-spondin-1 supplementation (ENR medium) formed as enteroids and were used as a positive control. Crypts grown in EN medium failed to form enteroids and were used as a negative control (Figure 1B).

The number of viable organoids and budding organoids were counted for each compound, and the viable and budding factors of each compound were calculated by correction with the viable and budding organoid numbers of the positive and negative controls. Thus, 295 primary hit compounds were selected as compounds with at least one of the viable and budding factors being positive (Supplementary Figure 1). The 295 compounds were reanalyzed under the same conditions, and 11 compounds with positive values for both the viable and budding factors were selected as secondary hits (Figure 1C and Supplementary Table 1). Finally, the 11 compounds were again tested at various concentrations, and one compound exhibited a concentration-dependent effect and consistently reproduced the effect, which was named RS-246204 (Figure 1D).

### Effects of RS-246204 on enteroid formation and growth

**Figure 2:**
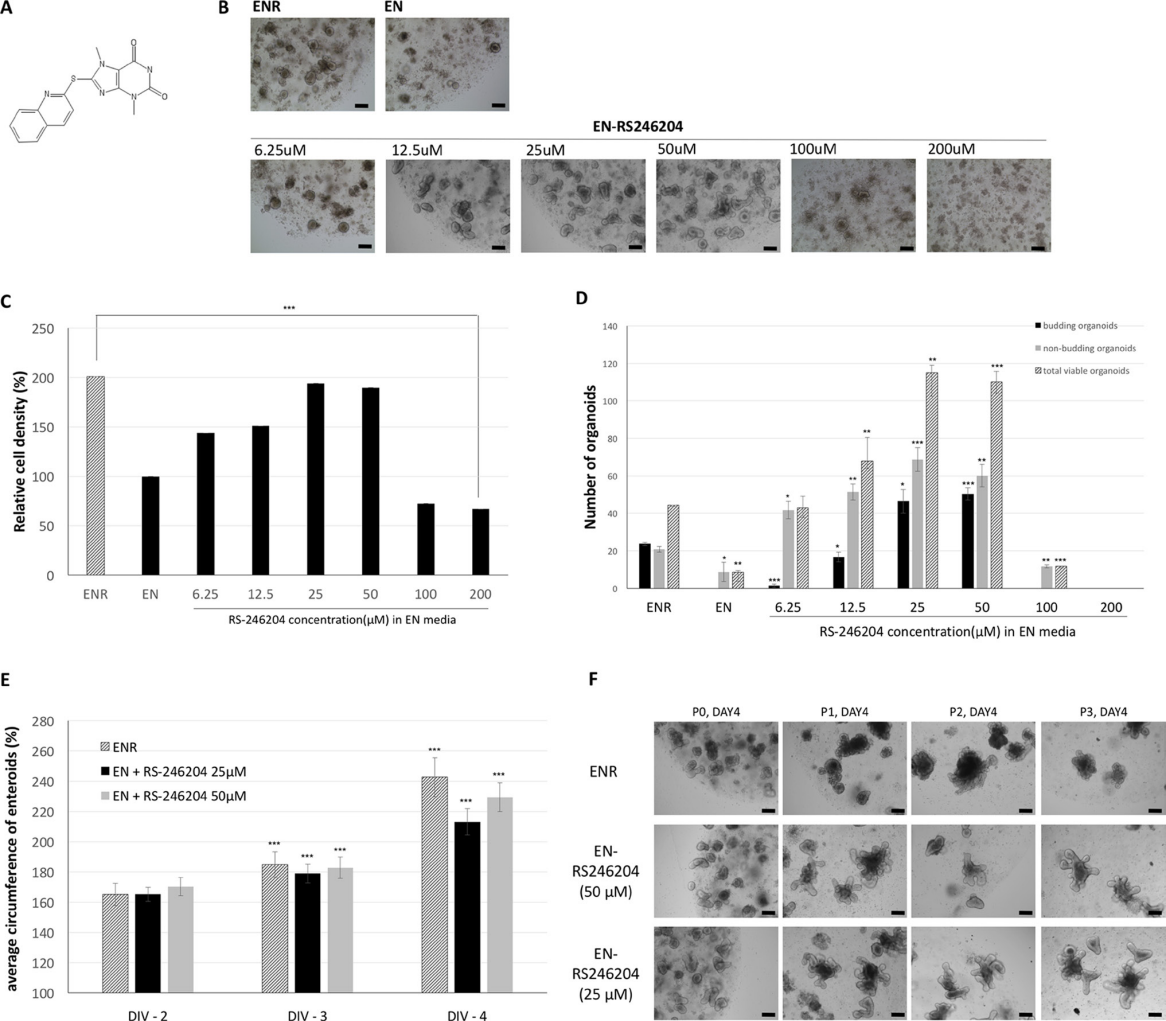
Effects of RS-246204 on enteroid formation and growth (**A**) The chemical structure of RS-246204. (**B**) Bright-field images of enteroids treated with various concentrations of RS-246204. Bars, 100 μm. (**C**) Relative cell density of the enteroids after 4 days of culturing in various concentrations of RS-246204 by the WST-1 assay. *n* = 6; ENR, 0 μM RS-246204 group, *n* = 4; 6.25 μM, 12.5 μM, 100 μM and 200 μM RS-246204 group, *n* = 10; 25 μM and 50 μM RS-246204 group. n.s.: not significance. (**D**) Count of budding and non-budding enteroids after 4 days of culturing with various concentrations of RS-246204. The total viable enteroid is the sum of the budding and non-budding; *n* = 3 samples per bar. (**E**) Time response graph for the circumference of the enteroids grown with R-spondin-1 or RS-246204. DIV refers to the day *in vitro*. *n* = 36; ENR group, *n* = 54; EN+25 μM RS-246204 group, *n* = 68; EN+50 μM RS-246204 group. (**F**) Representative images of the enteroids of each condition after subculture. Bars, 100 μm. Data are presented as the mean ± SEM, ^***^p < 0.001, ^**^p < 0.01, ^*^p < 0.05.

From the library screen, RS-246204, a 2-substituted purine, was identified to significantly increase mouse enteroid formation in growth medium without R-spondin-1 (Figure 2A). We examined the effect of RS-246204 on enteroid formation from various aspects and observed the concentration dependence of RS-246204. RS-246204 was added to EN media (EN-RS246204 medium) at 2-fold intervals from 6.25 μM to 200 μM, and the efficiency of organoid formation from the crypts was observed after 4 days of incubation. We found that the concentrations between 50 and 25 μM effectively induced the crypts to form enteroids. Above 200 μM, all crypts failed to become enteroids, and below 6.25 μM, the enteroids failed to form buds (Figure 2B). Next, when the WST-1 activity was measured to estimate the number of viable cells, it was confirmed that when 25 μM to 50 μM of RS-246204 was added, the estimated number of viable cells was similar to that of the ENR medium (Figure 2C). The small intestinal epithelium crypt-like structure in the enteroids is a bud, which mainly contains epithelium renewal-related cells such as intestinal stem cells and Paneth cells. Thus, an important indicator of a normal organoid is the presence of a bud, and we observed the RS-246204 treatment to determine whether buds formed in the cultured enteroids. At the 25 and 50 μM concentrations of RS-246204, the number of budded organoids was greatly increased, but no budded enteroids were observed below 6.25 μM or above 100 μM (Figure 2D). To track the growth rate of the organoids, we measured the length of the circumference of the enteroids. The organoids with RS-246204 grew at a rate similar to that of the enteroids on ENR media. (Figure 2E). It was confirmed whether the organoids could be cultured with RS-246204 in other organs than in the small intestine. However, it failed to propagate colonoids from the colon crypt, and RS-246204 could not replace R-spondin-1 (Supplementary Figure 2).

We searched for other compounds that could further promote enteroid growth besides RS-246204. In the first approach, seven compounds similar to that of RS-246204 with a Tanimoto coefficient of 0.9 or greater were analyzed as R-spondin-1 substitutes, and only two compounds (Pubchem CID: 8307286 and 16260909) could induce the budding and growth of enteroids from crypts (Supplementary Figure 3C, 3E). However, we failed to find compounds with a better efficiency than that of RS-246204. In the second approach, the chemical library of the Korean Chemical Bank was investigated to find a compound with a structure similar to that of RS-246204, and 79 compounds were selected and analyzed as R-spondin-1 substitutes. As a result, there were some compounds capable of inducing some budding and growth of enteroids, but no compounds could be found that had a better efficacy than that of RS-246204 (Supplementary Figure 4).

The organoids grown in the EN-RS246204 media could proliferate successfully even when subcultured at intervals of 5 days. Based on the above results, it was confirmed that various growth indexes such as formation, growth, budding, and passaging efficiency of enteroids in EN-RS246204 media containing RS-246204 are similar to that of ENR media containing R-spondin-1 and that the most effective concentrations were between 25 and 50 μM.

### Effects of RS-246204 on the differentiation of enteroids

**Figure 3:**
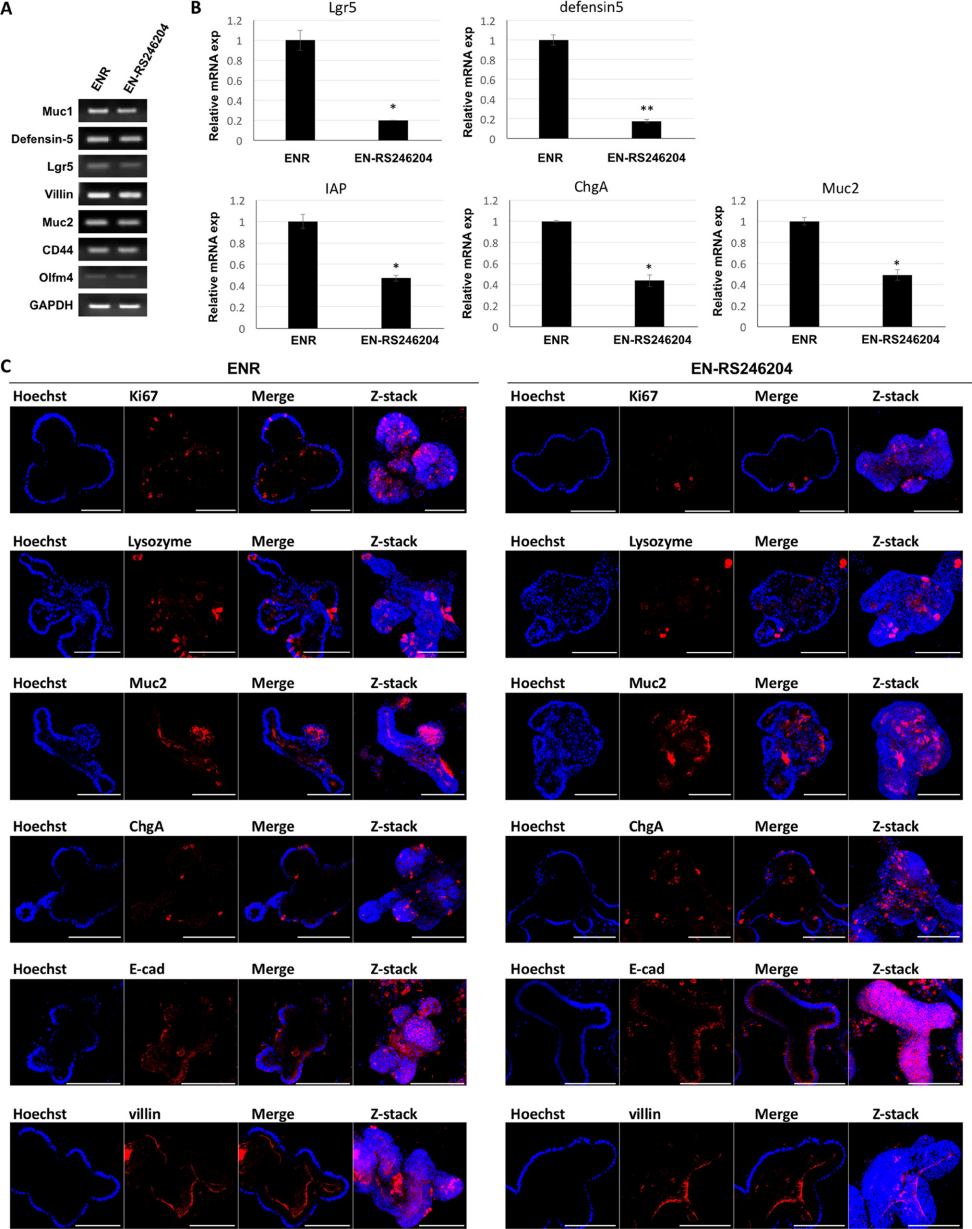
Effects of RS-246204 on the differentiation of enteroids (**A**) mRNA expression in enteroids grown in ENR or EN-RS-246204 was quantified by RT- PCR. (**B**) Relative mRNA expression in enteroids grown in ENR or EN-RS-246204 was quantified by qRT- PCR. Data are presented as the mean ± SEM; *n* = 2 samples per bar. (**C**) The expression of Ki67, Lysozyme, Mucin-2, Chromogranin A, E-cadherin and Villin in enteroids grown in ENR or EN-RS-246204 was examined by immunofluorescence. Bars, 100 μm.

Similar to the small intestinal epithelium, enteroids grown in ENR media are composed of intestinal stem cells, Paneth cells, enterocytes, enteroendocrine cells and goblet cells. We investigated whether enteroids cultured in EN-RS246204 media express lineage specific markers. We used Lgr5 for intestinal stem cells, mucin-1 and -2 (Muc-1, -2) for goblet cells, defensin-5 for Paneth cells, chromogranin A (ChgA) for enteroendocrine cells, and villin for brush boarder cells as lineage specific markers. RT-PCR was able to detect marker expression for all epithelial lineage cells in the EN-RS246204-grown enteroids similar to the ENR-grown enteroids (Figure 3A). The lineage marker expression was quantitatively analyzed by qRT-PCR. The EN-RS246204-grown enteroids showed lower mRNA expression levels than the ENR-grown enteroids (Figure 3B). Next, immunostaining was performed with lineage markers to confirm the spatial distribution of the specific cells in the enteroids. The EN-RS246204-grown enteroids showed similar expression patterns to that of the ENR-grown enteroids for all markers including Ki67, Lysozyme, Muc2, ChgA, E-cad and Villin (Figure 3C). Based on the relative amount of mRNA, the ratio of Lgr5 and Defensin5 in EN-RS246204-grown enteroids was reduced compared to the ENR-grown enteroids, and the ratio of Muc2, ChgA, and IAP was increased (Supplementary Figure 5). These results suggest that the enteroids grown in the RS-246204 based medium have a similar differentiation capacity as well as self-renewal capacity as those grown in the R-spondin-1 based medium.

### RS-246204 regulates intestinal stem cell proliferation on enteroid formation and growth

**Figure 4:**
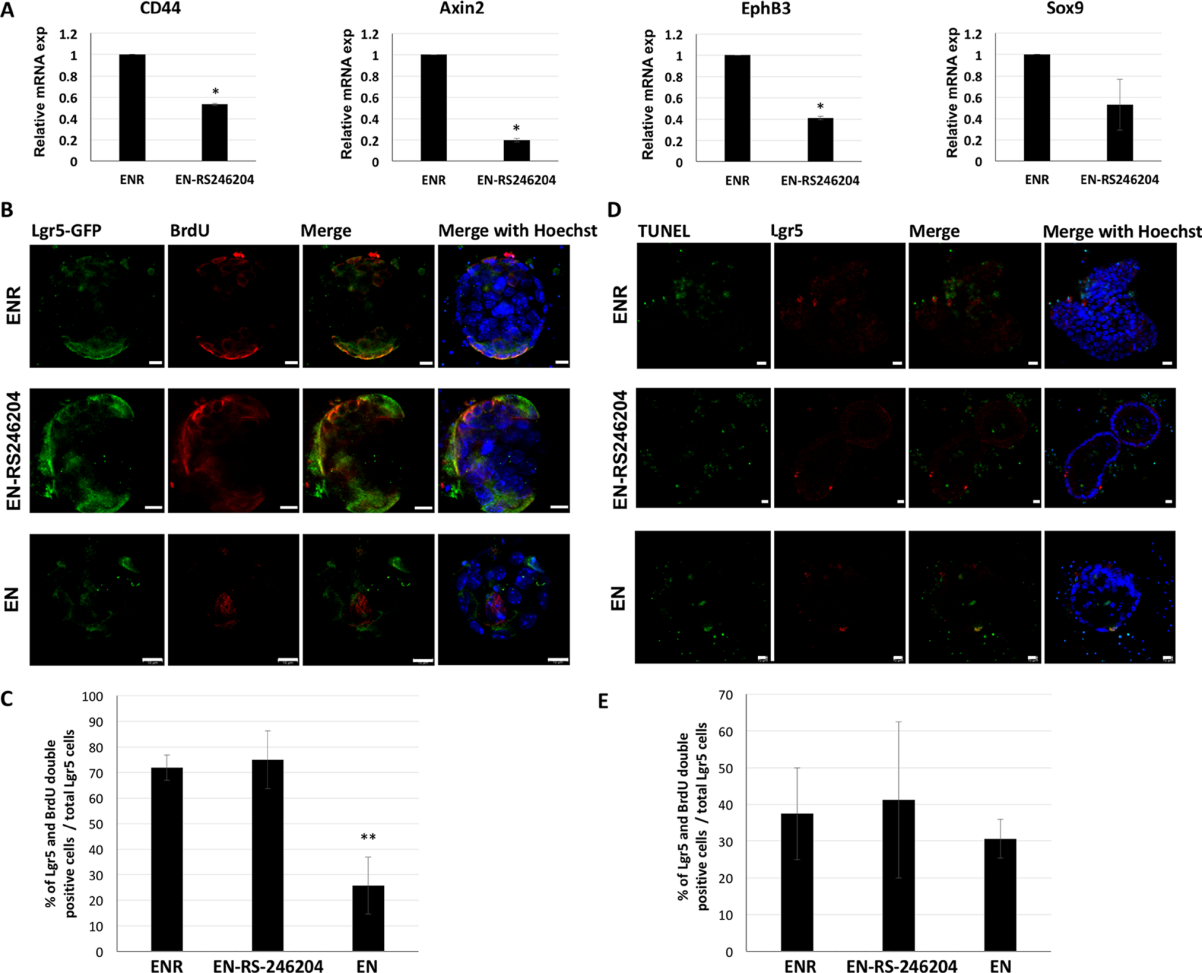
RS-246204 regulates intestinal stem cell proliferation on enteroid formation and growth (**A**) mRNA expression of Wnt target genes in ENR- or EN-RS-246204-grown enteroids was quantified by RT- PCR. (**B**) BrdU staining following 24 hours BrdU pulse labeling shows that BrdU positive cells were labeled with red fluorescence in enteroids grown in EN, ENR or EN-RS-246204. Lgr5 positive cells were labeled with green fluorescence, Bars, 10 μm. (**C**) The ratio of the BrdU and Lgr5 double positive cells to total Lgr5 positive cells per enteroid was quantified; *n* = 14; ENR group, *n* = 6; EN-RS246024 group, *n* = 10; EN group. (**D**) After 2 days of crypt seeding, TUNEL assay was performed to assess apoptosis. TUNEL positive cells were labeled with green and Lgr5 positive cells with red fluorescence, Bars, 10 μm. (**E**) The ratio of the TUNEL and Lgr5 double positive cells to total Lgr5 positive cells per enteroid was quantified; *n* = 4; ENR group, *n* = 4; EN-RS246204 group, *n* = 5; EN groupgfdz. Data are presented as the mean ± SEM, ^***^p < 0.001.

R-spondin-1 / Lgr5 signaling acts as a positive modulator of Wnt signaling and promotes the proliferation of intestinal stem cells, which plays a crucial role in the formation of enteroids. Therefore, to determine whether RS-246204 could acts as a positive regulator for Wnt signaling, mRNA level of the Wnt target genes, CD44, Axin2, EphB3 and Sox9, were measured in the EN-RS246204-grown enteroids. The EN-RS246204-grown enteroids were expressed the Wnt target genes, but lower levels than the ENR-grown enteroids (Figure 4A).

To confirm that RS-246204 affects proliferation of Lgr5 stem cells, BrdU positive cells were observed after BrdU pulse labeling for 1 day. Number of BrdU positive cells, and the ratio of BrdU and Lgr5 double positive cells to total Lgr5 positive cells was also similar in EN-RS246204- and ENR-grown enteroids. However, the number of BrdU positive cells and the ratio of BrdU and Lgr5 double positive cells to total Lgr5 positive cells were lower than those in EN-RS246204- and ENR-grown enteroids (Figure 4B, 4C). Next, to confirm that RS-246204 affects apoptosis of Lgr5 stem cells, the TUNEL assay was performed. As a result, EN, ENR, and EN-RS246204 showed similar in numbers of TUNEL positive cells and the ratio of TUNEL and Lgr5 double positive cells to total Lgr5 positive cells (Figure 4D, 4E). These results suggest that RS-246204 has effects as a positive regulator of Wnt signaling and an inducer of proliferation of Lgr5 stem cells similar to R-spondin-1.

### Functional analysis of EN-RS246204-grown enteroids

**Figure 5:**
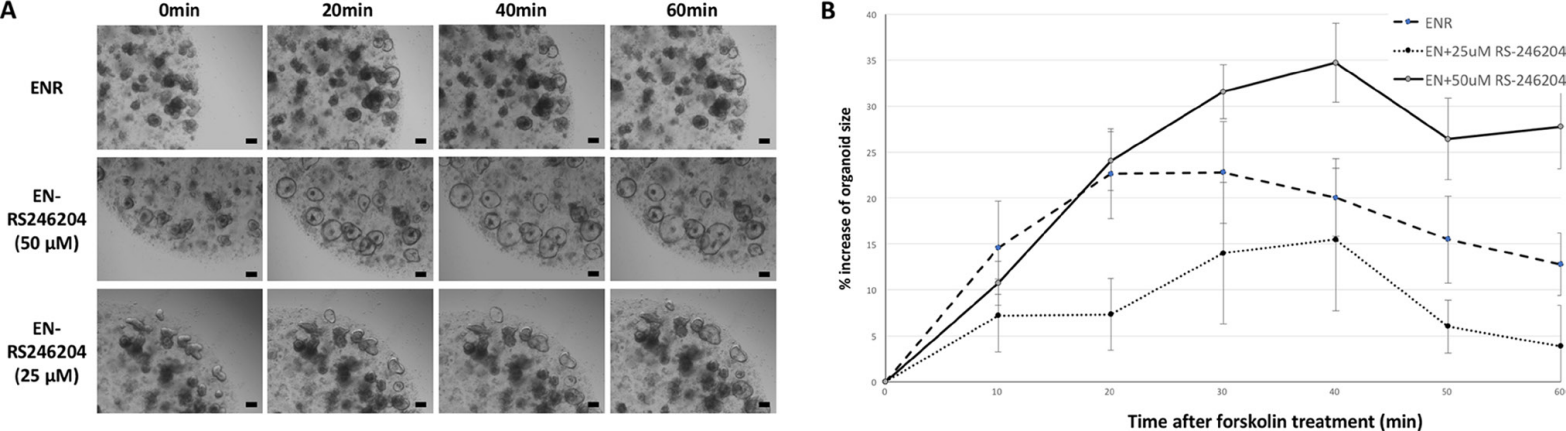
Functional analysis of the EN-RS-246204-grown enteroids by the forskolin induced swelling assay (**A**) Morphological changes of the enteroids over time after treatment with forskolin. Bars, 100 μm. (**B**) Time response graph for the circumference of the enteroids after treatment with forskolin. *n* = 10; ENR group, *n* = 6; EN+25 μM RS-246204 group, *n* = 11; EN+50 μM RS-246204 group. Data are presented as the mean ± SEM.

A cystic fibrosis transmembrane conductance regulator (CFTR) is a chloride channel expressed in intestinal epithelial cells which induces the secretion of electrolytes and fluid [21]. When normally functioning organoids are treated with forskolin, a CFTR agonist, electrolytes and water are secreted into the lumen of the organoids resulting in the swelling of the organoids [22]. We performed a forskolin induced swelling assay to confirm that the enteroids cultured in EN-RS246204 have a normal CFTR activity. The ENR-grown enteroids started to swell immediately after the forskolin treatment becoming the largest at 40 minutes and then slightly decreased. EN-RS246204-grown enteroids also showed forskolin-induced swelling at a similar rate as the ENR-grown enteroids (Figure 5A, 5B). This result shows that the EN-RS246204-grown enteroids can perform CFTR mediated electrolyte and water secretion functions normally.

**Figure 6:**
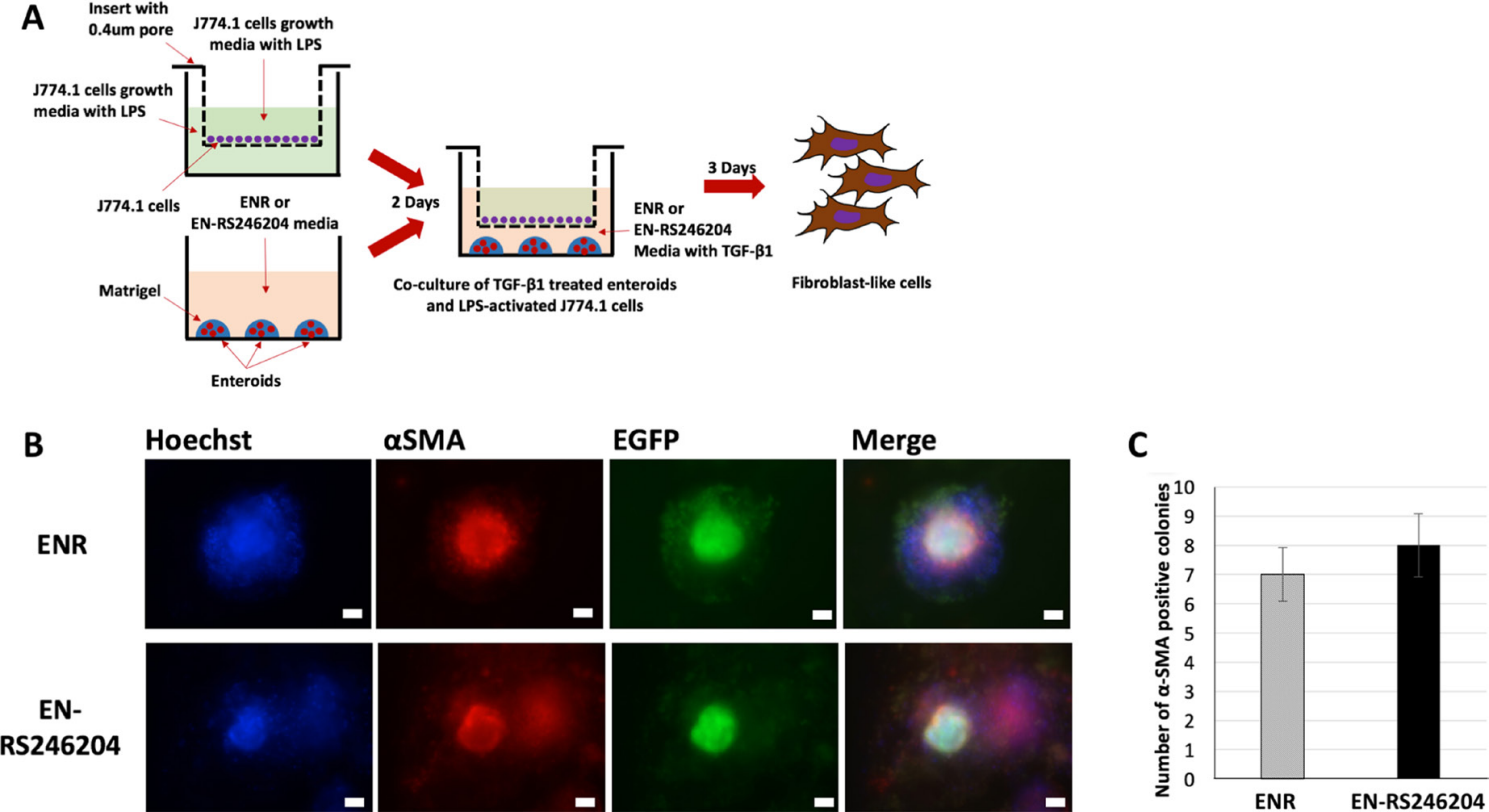
Functional analysis of the EN-RS246204-grown enteroids by production of the OEMT model (**A**) A schematic illustration of the OEMT model with the RS-246204-grown enteroids co-cultured with J774.1 cells. (**B**) α-SMA and EGFP expression of the OEMTs generated using RS-246204-grown enteroids. Bars, 50 μm. (**C**) Number of α-SMA positive colonies of OEMT derived from the enteroids grown in ENR or EN-RS246204 media. respectively. Data are presented as the mean ± SEM; *n* = 4 samples per bar.

Type 2 epithelial to mesenchymal transition (EMT) is known as one of the key mechanisms of intestinal fibrosis [23]. In our previous study, we generated an organoid-based EMT model (OEMT model) based on our finding that TNF-α and TGF-β synergistically induce type 2 EMT in enteroids [4]. To confirm that the enteroids grown with RS-246204 can also make an OEMT model, we established a three-dimensional co-culture system in which TGF-β1 treated enteroids derived from CAG-EGFP mice and LPS-stimulated J774.1 cells, a murine macrophage cell line, were grown on a 0.4 μm pore size transwell (Figure 6A). When the enteroid derived colonies were immunostained for α-SMA, a marker for mesenchymal cells, α-SMA expression in nearly all the mesenchymal like colonies was observed in the enteroids grown with ENR or EN-RS246204 (Figure 6B). All α-SMA positive cells have EGFP expression, which means that α-SMA positive cells are derived from the enteroids but not from the J774.1 cells (Figure 6C). The above results show that the RS-246204-derived enteroids can be used for the forskolin induced swelling assay and OEMT modeling. The RS-246204-derived enteroids can successfully produce the forskolin induced swelling and the OEMT model, which can reproduce the *in vivo* physiology and be used for drug screening and pathogenesis.

### Regenerative effects of RS-246204 in damaged intestinal epithelium

**Figure 7:**
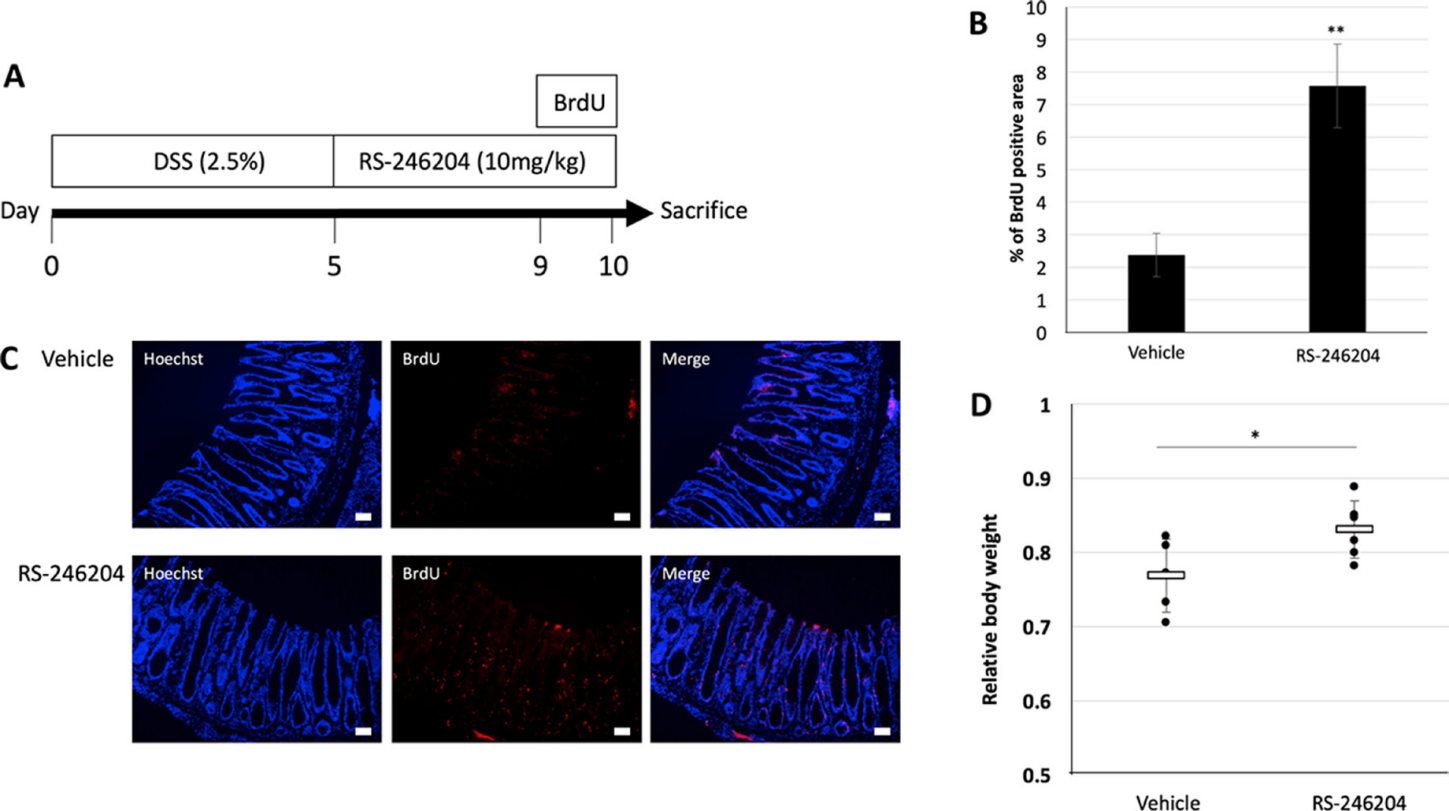
Regenerative effects of RS-246204 in the DSS induced colitis model (**A**) Schedule of the experiment. (**B**) The relative body weight of the DSS induced colitis models treated with vehicle and RS-246204. *n* = 5; vehicle group, *n* = 6; RS-246204 group. (**C**) The expression levels of BrdU in the large intestinal epithelium were compared between the vehicle and RS-246204 groups. Bars, 50 μm. (**D**) The area of the BrdU-positive cells per high-power field was counted. *n* = 10; vehicle group, *n* = 15; RS-246204 group. Data are presented as the mean ± SEM; ^**^p < 0.01, ^*^p < 0.05.

In intrinsic stem cells, the R-spondin-Lgr5 axis enhances Wnt signaling and is involved in self-renewal. It was shown that when R-spondin-1 was administered to mice that had intestinal damage induce by 5-FU, the intestinal damage decreased, and the survival rate increased [24]. In our results, RS-246204 can induce enteroid formation without R-spondin-1 suggesting that administrating RS-246204 to intestinal epithelium-deficient mice may promote regeneration of the intestinal epithelium. To confirm this hypothesis, we administered 2.5% DSS for 5 days to induce inflammation-mediated damage to the intestinal epithelium, and then, RS-246204 was administered to observe the regenerative effects on the intestinal epithelium (Figure 7A). In DSS induced colitis, body weight is an important indicator of disease severity. The RS-246204-treated group had a significantly higher body weight than that of the vehicle-only group (Figure 7B). To observe the regeneration of the intestinal epithelium, BrdU was administered 1 day before sacrifice, and the number of BrdU positive cells in the large intestinal epithelium was counted. We observed that BrdU positive cells increased more than 3 times when RS-246204 was administered (Figure 7C, 7D). These results suggest that RS-246204 may be able to support intestinal stem cell proliferation and regeneration of intestinal tissue *in vivo*.

## DISCUSSION

The addition of EGF, Noggin, and R-spondin-1 in intestinal organoid growth medium is essential for intestinal organoid induction and expansion. In particular, without the addition of R-spondin-1, no primary intestinal organoid formation is observed (Figure 2B). In this study, 8,364 compounds in a chemical library were used to identify one compound capable of culturing intestinal organoids without R-spondin-1 which was named RS-246204. Furthermore, the enteroids cultured with EN-RS246204 had a similar growth (Figure 2D–2E), spatial organization (Figure 3) and intestinal specific function (Figures 5, 6) as the intestinal enteroids cultured with ENR. This is the first report to discover substitute compounds for R-spondin-1 in intestinal organoid cultures.

The R-spondin / Lgr5 / β-catenin module is known to be a positive regulator of the Wnt signal strength, which is involved in the self-renewal of intestinal stem cells and maintenance of tissue homeostasis [25]. In our experiment, when CHIR99021, the Wnt signaling activator, was added without R-spondin-1 or RS-246204, it failed to form enteroids from the crypts. However, it was possible to maintain by adding CHIR99021 without R-spondin-1 or RS-246204 in the secondary enteroids. Additionally, RS-246204 and CHIR99021 must be put together, but initial organoid formation from the crypt is possible without this R-spondin-1. These results suggest that the enteroid formation capacity by the R-spondin / Lgr5 signaling pathway involves other signaling pathways associated with the survival of intestinal stem cells besides β-catenin mediated signaling. RS-246204 is likely to be involved in the regulation of another pathway other than β-catenin mediated signaling by the R-spondin / Lgr5 module. Vieira GC, et al. reported that pro-survival signaling is involved in the Wnt independent Lgr5 / Ras / MEK / ERK pathway in Neuroblastoma cells [26]. Wnt independent apoptosis in neuroblastoma cell-lines is accompanied by greatly diminished phosphorylation of MEK1/2 and ERK1/2 and an increase in BimEL, an apoptosis facilitator downstream of ERK. Wnt-dependent signaling has an important role in enteroid growth by maintaining the stemness of the Lgr5+ stem cells, and Wnt-independent signaling involved in anti-apoptosis has a role in the formation of enteroids. Based on these results, one possible explanation is that R-spondin-1 activates both Wnt-dependent and Wnt-independent signaling, while RS-246204 activates only Wnt-independent signals, and CHIR99021 activates only Wnt-dependent signals. RS-246204 will help identify novel functions of the R-spondin / Lgr5 module.

There was a difference in the type of cells constituting the enteroids grown in the R-spondin-1-based medium and in RS-246204-based-medium. The expression of Lgr5 and Defensin5 in EN-RS246204-grown enteroids was reduced compared to the ENR-grown enteroids, and the expression of Muc2, ChgA, and IAP was increased (Supplementary Figure 5). Wnt signaling is a key factor in balancing the division and differentiation of intestinal stem cells [27]. R-spondin-1 enhances Wnt signaling and predominantly regulates intestinal stem cell division. RS-246204 showed to have less efficacy in activating Wnt signaling than R-spondin-1 (Figure 4A). These results demonstrate the mechanism by which the proportion of intestinal stem cells and differentiated cells in RS-246204-grown enteroids are changed compared to R-spondin-1-grown enteroids.

To find compounds with a better potency for enteroid formation compared to RS-246204, we tested available compounds with a structure similar to RS-246204 (Supplementary Figures 3 and 4). Some compounds showed efficacy for intestinal organoid formation but did not show a superior effect on enteroid formation compared to RS-246204. Further analysis of the structural similarities between RS-246204 and effective compounds will provide clues to find more efficient compounds or binding partners and to clarify the mechanism of action for RS-246204.

We hypothesized that RS-246204 could promote intestinal epithelium regeneration *in vivo* based on the R-spondin-1 equivalent effects in enteroid formation. RS-246204 significantly increased BrdU+ cells and body weight in the DSS induced colitis model (Figure 6). These results suggest that RS-246204 activates the R-spondin dependent pathway in Lgr5 stem cells, thereby enhancing proliferation or the anti-apoptotic effect, promoting regeneration of the intestinal epithelium and reducing the disease severity. Clarifying the therapeutic effects of RS-246204 will enable the development of new treatments for de-epithelialized diseases in the GI tract.

Despite great advancements in generating organoid culture models recapitulating the human *in vivo* physiology, generating organoids in the R-spondin-1 based culture medium is not available in many laboratories due to the high costs and long duration as well as the advanced technology required. The enteroids formed by the methods described in this study can be easily generated at low costs using RS-246204 and intestinal crypts. It was confirmed that the RS-246204 based enteroids could be used to make the OEMT model as well as for forskolin induced swelling similar to the R-spondin-made enteroids, thus confirming no functional difference.

The EN-RS-246204-grown enteroids were successful in inducing the OEMT model as well as in inducing swelling by forskolin. These results mean that there is no functional difference with the ENR-grown enteroids (Figure 6). The possibility to culture enteroids at low cost can economically enable high throughput screening to find drugs such as inhibiting EMT for intestinal fibrosis or activating CFTR for cystic fibrosis.

In conclusion, during a chemical library screen, RS-246204 was found as a R-spondin-1 substitute for organoid cultures. This compound could be used to develop a cost-efficient culturing method of intestinal organoids as well as to explore Lgr5 signaling, intestinal stem cell physiology and therapeutics for GI tract diseases.

## MATERIALS AND METHODS

### Animals

The CHA University Institutional Animal Care and Use Committee approved all animal studies. All efforts were made to minimize animal suffering and to reduce the number of animals used. Male C57Bl/6 mice were obtained from Orient Bio (Seongnam, Korea) and maintained in the animal facility of CHA University under 12-h light/dark cycle with food and water available *ad libitum*.

### Preparation of mouse small intestinal organoids

Preparation of mouse small intestinal organoids was done with 8- to 10-week-old C57Bl/6 mice weighing 20∼25 g each. Mouse small intestines were removed after sacrifice by cervical dislocation. Crypts were then isolated by treatment with Gentle Cell Dissociation Reagent (StemCell Technologies, Cambridge, MA) and filtered with a 70 μm cell strainer. Isolated crypts were mixed with Matrigel (BD Biosciences, Franklin Lakes, NJ) at a ratio of 1:1, and the mixture was seeded in test plates. After polymerization of the Matrigel by incubating at 37°C for 10 minutes, organoid culture medium was added. The basic composition of the organoid culture medium (referred to as EN medium) is as follows: Advanced DMEM/F-12 (Life Technologies, Gaithersburg, MD), HEPES Buffer (Life Technologies), GLUTAMAX -I supplement (Life Technologies), Penicillin-Streptomycin Solutions (JBI, Daejeon, Korea), N-Acetyl-L-cysteine (Sigma-Aldrich, St. Louis, MO), B-27 Serum-Free Supplement (Life Technologies), N-2 Supplement (Life Technologies), Animal-Free Recombinant Murine EGF (PeproTech, Rocky Hill, NJ), Recombinant Murine Noggin (PeproTech), CHIR99021 (Sigma-Aldrich), and Thiazovivin (Sigma-Aldrich).

### Screening procedure

Crypts derived from the small intestine of C57Bl/6 mice weighing 20∼25 g each were mixed in Matrigel (BD Biosciences), and 10 μl aliquots were seeded into 96 multi-well plates. Six wells were used for the positive and negative controls, respectively. Positive controls were grown in EN medium containing 10% R-spondin-1 conditioned medium (referred to as ENR medium). Negative controls were grown in EN media, which did not contain either R-spondin-1 or screening compounds. The experimental groups were grown in EN medium containing 50 μM of a screening compound. A total of 8,364 compounds in the representative library of the Korea Compound Bank were tested. After incubation for 4 days at 37°C in a humidified incubator (5% CO_2_ in air), images of the grown organoids were obtained by light microscopy (Olympus CKX41, Center Valley, PA). To evaluate the effect of each compound, the viable factor and budding factor for each compound were calculated as follows.

Viable factor = (No. of viable organoids in EN media containing one screening compound - No. of viable organoids in EN media) / No. of viable organoids in ENR media Budding factor = (No. of budding organoids in EN media containing one screening compound - No. of budding organoids in EN media) / No. of budding organoids in ENR media

### Immunocytochemistry

Organoids were washed with ice-cold DPBS until the Matrigel was disintegrated. The free-floating organoids were fixed with 4% paraformaldehyde, permeabilized with 0.1% Tween-20 and 0.2% Triton-X100 in PBS buffer and then blocked with 5% normal goat serum in DPBS to reduce non-specific binding. Samples were covered with primary antibodies at 4°C overnight. The next day, the samples were covered with secondary antibodies at room temperature for 2 h and counterstained with Hoechest33342 (Sigma-Aldrich). Antibodies used for the staining were as follows: mouse anti-Mucin-2 (Santa Cruz Biotechnology, sc-15334 , Santa Cruz, CA), rabbit anti-Ki67 (Abcam, ab16667, Cambridge, MA), mouse anti-Chromogranin A (Santa Cruz Biotechnology, sc-393941), rabbit anti-Lysozyme (Diagnostic Biosystems, RP028, Fremont, CA), mouse anti-Villin (Santa Cruz Biotechnology, sc-58897), mouse anti-E-cadherin (Santa Cruz Biotechnology, sc-8426), α-SMA (Biolegend, 904601, San Diego, CA), mouse anti-GFP (Santa Cruz Biotechnology, sc-8334), rabbit anti-Lgr5 (Abgent, AP2745d, San Diego, CA), goat Alexa Flour 488 conjugated anti-rabbit IgG (Life Technologies, A11034) and goat Alexa Flour 594 conjugated anti-mouse IgG (Life Technologies, A11032). Stained organoids were finally mounted onto glass slides (Marienfeld, Lauda-Koenig-Shofen, Germany) and examined with a Leica TCSSP5II confocal microscope (Leica, Heidelberg, Germany).

### Forskolin assay

Using enteroids 4 days after seeding, the culture medium was removed and replaced with an organoid culture medium containing 5 μM forskolin (Sigma-Aldrich). To analyze the morphological changes, images were taken at intervals of 10 minutes for 1 hour immediately after the forskolin treatment by light microscopy (Olympus). The perimeter of the organoids was measured with the eXcope software (DIXI optics, Daejeon, Korea).

### TUNEL assay

Crypts isolated from a small intestine were seeded into 8 multi-well cell culture slides and cultured. For days after the incubation, cell apoptosis were assayed with the DeadEnd™ Fluorometric TUNEL System (Promega, Mannheim, Germany). The organoids were fixed with 4% paraformaldehyde, permeabilized with 0.2% Triton-X100 in PBS buffer and then equilibrate with equilibration buffer for 10 minutes. After remove the equilibration buffer, add the nucleotide mix and rTdT enzyme in equilibration buffer and incubate at 37°C for 1 hour to label the broken DNA strand with fluorescein-12-dUTP. The reaction was stopped with 2X SSC and counterstained with Hoechst 33342. Stained organoids were mounted and examined with a Leica TCSSP5II confocal microscope. (Leica)

### 5-Bromo-2’-deoxyuridine (BrdU) pulse-chase labelling

Organoids from small intestinal crypt were cultured for 3 days, and then BrdU (Sigma-Aldrich) was added to the culture medium at 3 μg/mL. After 24 h, culture media was removed and fixed with 4% paraformaldehyde. 2N HCl was treated at room temperature for 30 min to expose BrdU labeled epitopes. Cells were blocked with 5% normal goat serum in PBS for 1 h followed by incubation with 2 µg/mL of rat monoclonal anti-BrdU antibody (Novus Biologicals, NB500-169, Littleton, CO) at 4°C. After 12 hours, cells were incubated with goat Alexa 594-conjugated anti-rat IgG antibody (Life Technologies) at room temperature for 2 hours. Counterstain were used to Hoechst33342 (Sigma-Aldrich).

### Quantitative reverse transcriptase-polymerase chain reaction (qRT-PCR) analysis

The organoids were treated with Cell Recovery Solution (Corning Incorporated, Corning, NY) to remove the Matrigel, and total RNA was extracted with the MagListo™ 5M Cell Total RNA Extraction Kit (Bioneer, Daejeon, Korea). Total RNA extraction was done following the manufacturer’s instructions. RNA was synthesized to cDNA with AccuPower® RocketScript™ Cycle RT Premix (Bioneer). qRT-PCR was performed with SYBR Premix Ex Taq (Takara, Tokyo, Japan) and Thermal Cycler Dice Real Time System III (Takara). The reaction was performed as follows: denaturation at 95°C for 5 min. followed by 30 cycles of denaturation at 95°C for 10 s, annealing at 57°C for 15 s, and extension at 72°C for 20 s with a final extension step at 72°C for 5 min. The primer pairs used for the qRT-PCR were as follows: a primer pair for mouse GAPDH, forward 5′-CAGTATGACTCCACTCACGG-3′and reverse 5′-GTGAAGACACCAGTAGACTCC-3′; a primer pair for mouse Lgr5, forward 5′-GACGCTGGGTTATTTCAAGTTCAA-3′ and reverse 5′-CAGCCAGCTACCAAATAGGTGCTC-3′; a primer pair for mouse Defensin-5, forward 5′-AGGCTGATCCTATCCACAAAACAG-3′ and reverse 5′-TGAAGAGCAGACCCTTCTTGGC-3′; a primer pair for mouse Intestinal Alkaline phosphatase, forward 5′-GCCTATCTCTGTGGGGTCAA-3′ and reverse 5′-TTTCTTGGCACGGTACATCA-3′; a primer pair for mouse Chromogranin A, forward 5′-AAGGTGATGAAGTGCGTCCT and reverse 5′-GGTGTCGCAGGATAGAGAGG-3′; a primer pair for mouse Muc-2, forward 5′-ATGCCCACCTCCTCAAAGAC-3′ and reverse 5′-GTAGTTTCCGTTGGAACAGTGAA-3′; a primer pair for mouse CD44, forward 5′-CACATATTGCTTCAATGCCTCAG-3′ and reverse 5′-CCATCACGGTTGACAATAGTTATG-3′; a primer pair for mouse Axin2, forward 5′-GCAGCAGATCCGGGAGGATGAA-3′ and reverse 5′-GATTGACAGCCGGGGGTCTTGA-3′; a primer pair for mouse EphB3, forward 5′-AAGAGACTCTCATGGACACGAAATG-3′ and reverse 5′-ACTTCCCGCCGCCAGATG-3′; a primer pair for mouse Sox9, forward 5′-TACGACTGGACGCTGGTGCC-3′ and reverse 5′-CCGTTCTTCACCGACTTCCTCC-3′; a primer pair for mouse Muc-1, forward 5′-CAATGGCACCTCAGTCAC-3′ and reverse 5′-TTGTGGTCTGGAATGATAGC-3′, and a primer pair for mouse villin, forward 5′-GACGTTTTCACTGCCAATACCA-3′ and reverse 5′-CCCAAGGCCCTAGTGAAGTCTT-3′.

### WST-1 activity assay

Crypts isolated from a mouse small intestine were seeded into 96 multi-well plates and cultured. Four days after the incubation, cell viability was assayed with the EZ-Cytox assay kit (Daeil Lab Service, Seoul, Korea) by adding water soluble tetrazolium salt to each well and then were incubated for another 3 hours. After the 3-hour incubation, only the medium of each well was transferred to new wells on another 96-well plate, and the absorbance was measured at a wavelength of 450 nm with a VersaMax ELISA Microplate Reader (Molecular Devices, CA, USA).

### Production of the enteroid-based epithelial to mesenchymal transition (OEMT) model

The murine macrophage cell line J774.1 (Korean Cell Line Bank, Seoul, Korea) was seeded into a 0.4 μm-pore-size transwell. The next day, the J774.1 cells were treated with 2 μg/ml lipopolysaccharide (LPS) from Escherichia coli O111:B4 (Sigma-Aldrich) to induce pro-inflammatory cytokines. For OEMT induction, the transwell with the J774.1 cells were transferred to the wells with the organoids treated with 8 ng/ml of recombinant Mouse TGF-β1. After 5 days of TGF-β1 treatment, immunocytochemistry was performed to analyze the efficiency of the OEMT.

### Induction of DSS-induced colitis and administration of RS-246204

Seven-week-old C57Bl/6 mice were given a 2.5% dextran sulfate sodium (DSS) (MP Biomedicals, Aurora, OH) treatment in drinking water for 5 days. RS-246204 (10 mg/kg in DMSO) was administered intraperitoneally at the end of the DSS exposure. The RS-246204 treatment was repeated every 24 h for 5 days. Daily assessments of disease activity were performed including measuring body weight and evaluating stool consistency. The mice were sacrificed the next day after the last injection of RS-246204. BrdU (50 mg/kg in saline) (Sigma-Aldrich) was administered intraperitoneally 24 hours before sacrifice.

### Immunohistochemistry

Mouse colons were removed and post-fixed in 4% formaldehyde overnight at 4°C and then cryoprotected in 30% sucrose (Sigma-Aldrich) in phosphate-buffered saline (PBS). Mouse colons were then stored at -80°C in an optimal cutting temperature (OCT) solution (Sakura Finetek, Torrance, CA, USA) and finally sectioned at a thickness of 20 μm using a cryostat (Shandon Cryostat, Thermo Fisher Scientific, Waltham, MA). To perform the BrdU staining, colon sections were incubated in 2 N HCl (Sigma-Aldrich) at 37°C for 20 min. Sections were then blocked with 10% goat serum (Biolegend) in PBS for 1 h followed by incubation with 2 µg/mL of a rat monoclonal anti-BrdU antibody (Biolegend) at 4°C for 12 h and then with 2 µg/mL of a goat Alexa 594-conjugated anti-rat IgG antibody (Life technologies) for 2 hours at room temperature. Sections were examined with a fluorescence microscope (Carl Zeiss, Göttingen, Germany).

### Statistical analysis

Differences between two groups were assessed by the Student’s *t*-test. Three or more groups were compared with one-way ANOVA followed by the Student-Newman-Keuls post hoc test. Differences were considered statistically significant at *p* < 0.05.

## SUPPLEMENTARY MATERIALS FIGURES AND TABLE


