# Supplementary material for: Effects of a small molecule R-spondin-1 substitute RS-246204 on a mouse intestinal organoid culture
Source: Oncotarget. 2017 Dec 26;9(5):6356–68. doi: 10.18632/oncotarget.23721 (PMC5814218; doi:10.18632/oncotarget.23721)
Supplement: Supplementary file 1 [file oncotarget-09-6356-s001.pdf]

Effects of a small molecule R-spondin-1 substitute RS-246204 on a mouse intestinal organoid culture

SUPPLEMENTARY MATERIALS

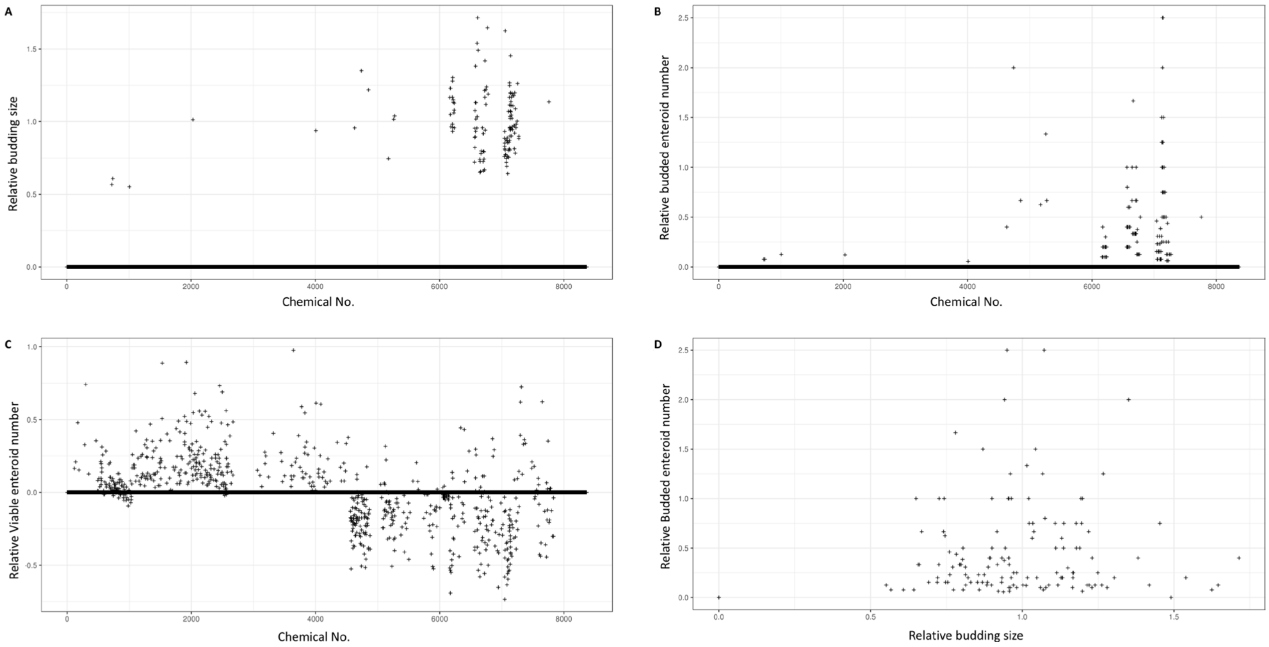

Supplementary Figure 1: Primary screening results to find a small molecule R-spondin-1 substitute. (**A**) Screening results based on relative budding size, (**B**) Screening results based on relative budded enteroid number, (**C**) Screening results based on relative viable enteroid number, (**D**) Plot for relative budding size and budded enteroid number.

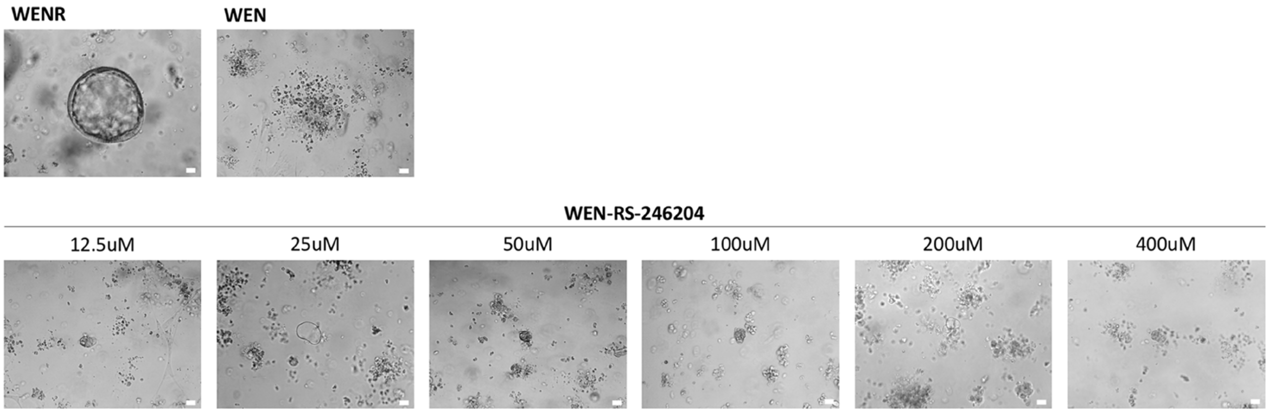

Supplementary Figure 2: Effects of RS-246204 on colonoid formation and growth. Bright-field images of colonoids treated with various concentrations of RS-246204. Bars, 100 μm.

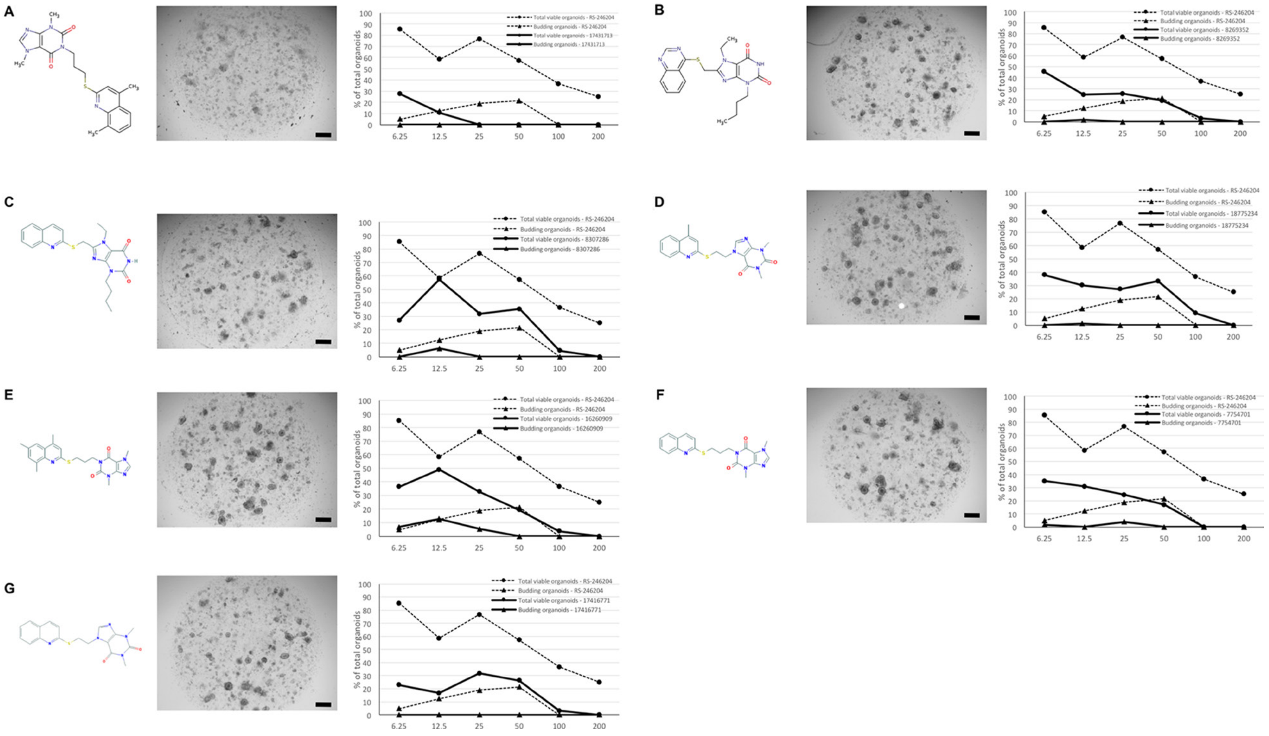

Supplementary Figure 3: Efficacy analysis for seven compounds with tanimoto coefficient of 0.9 or higher for RS-246204. For each compound panel, the chemical structure on the left, the representative photograph on the middle, and the test result on the right. (**A**) PubChem CID: 17431713, (**B**) PubChem CID: 826952, (**C**) PubChem CID: 8307286, (**D**) PubChem CID: 18775234, (**E**) PubChem CID: 16260909, (**F**) PubChem CID: 7754701, (**G**) PubChem CID: 171671.

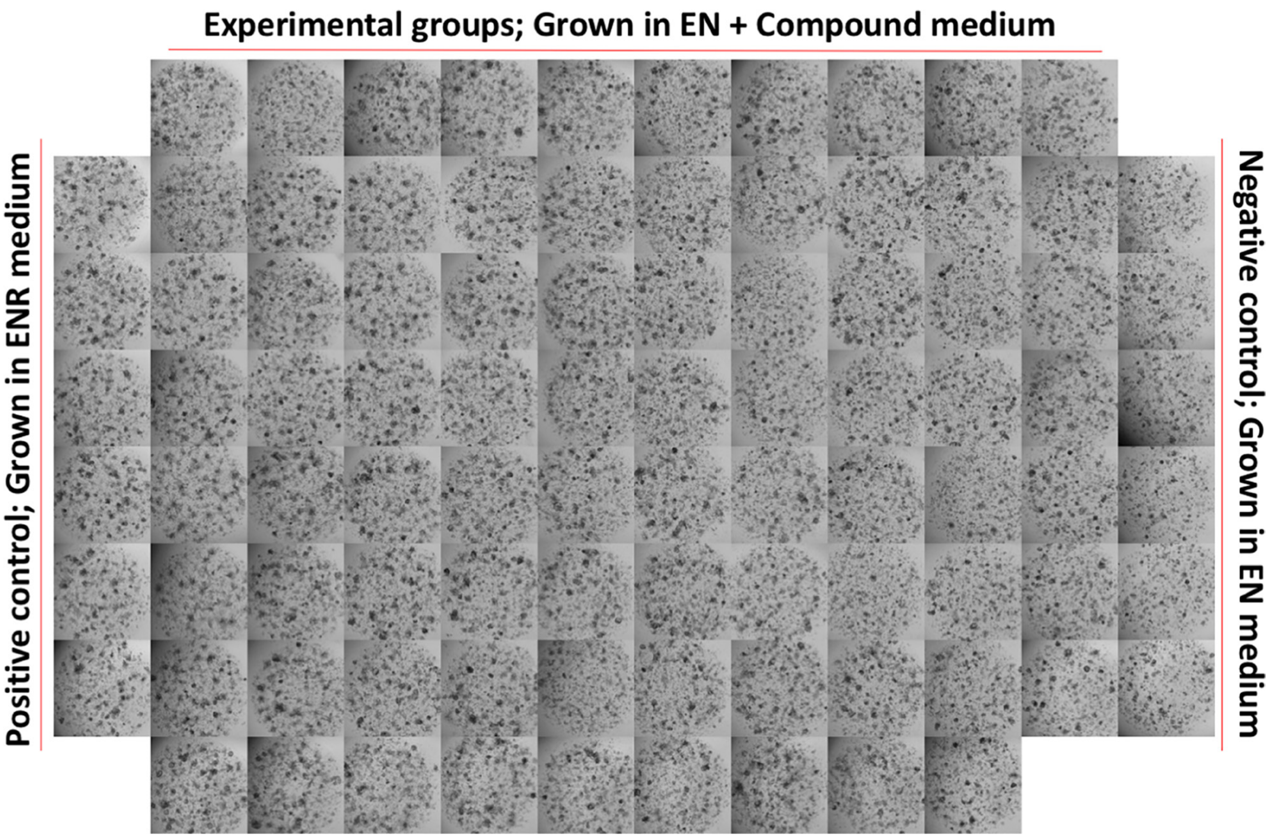

Supplementary Figure 4: Efficacy test for RS-246204-like compounds based on the chemical library of the Korean Chemical Bank. In order to confirm the effect of replacing 79 similar compounds with R-spondin, it was confirmed whether the compound could generate enteroids in the crypts.

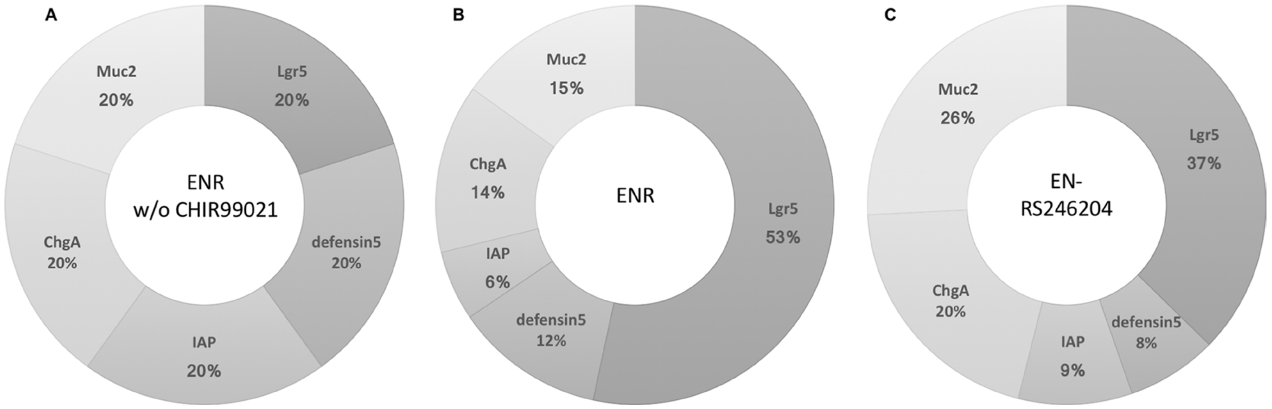

Supplementary Figure 5: Analysis of cellular composition by components of enteroid growth media. In order to investigate how the cellular composition of enteroid differs according to the media components, the proportion of each cell marker in the total is determined based on the amount of relative mRNA. Results from enteroids grown with ENR without CHIR99041 (**A**), ENR (**B**) and EN-RS246204 (**C**)

Supplementary Table 1: Detailed results for the efficacy of 11 compounds selected as primary hits

Chemial No.	Plate No.	Chemical ID	Chemical Position	Budding no	Budding size	Budding No. ratio	Budding size ratio	Budding No. ratio x Budding size ratio	Viable No	Viable No - Negative No	Viable No. ratio
2030	026	246204	U-001026-F06	4	516.8	0.12	1.01	0.123	46	45.83	0.548
4009	051	229510	U-001051-A04	2	441.7	0.056	0.94	0.052	46	45.83	0.614
6209	078	228585	U-001078-A09	1	384.8	0.1	0.96	0.096	128	12.33	0.097
6576	083	9855	U-001083-H04	1	508.4	0.2	1.13	0.226	86	10	0.099
6577	083	17349	U-001083-A05	2	621.3	0.4	1.38	0.553	106	30	0.297
6579	083	10094	U-001083-C05	2	419.3	0.4	0.93	0.373	77	1	0.010
6585	083	22518	U-001083-A06	3	508.0	0.6	1.13	0.678	102	26	0.257
6656	084	22723	U-001084-H04	1	390.4	0.33	0.66	0.219	87	3.33	0.036
6713	084	16781	U-001084-A12	3	441.1	1	0.74	0.742	94	10.33	0.112
7201	091	62921	U-001091-A03	2	365.18	0.12	0.81	0.102	98	10.33	0.086
7273	091	62853	U-001091-A12	2	403.4	0.12	0.90	0.112	105	17.33	0.144
